# Relevance of Fatty Acids to Sperm Maturation and Quality

**DOI:** 10.1155/2020/7038124

**Published:** 2020-02-05

**Authors:** Giulia Collodel, Cesare Castellini, Jetty Chung-Yung Lee, Cinzia Signorini

**Affiliations:** ^1^Department of Molecular and Developmental Medicine, University of Siena, Policlinico Le Scotte, Viale Bracci, 14, 53100 Siena, Italy; ^2^Department of Agricultural, Food and Environmental Sciences, University of Perugia, Borgo XX Giugno, 74, 06121 Perugia, Italy; ^3^School of Biological Sciences, The University of Hong Kong, Hong Kong

## Abstract

Almost 50% of infertility cases are associated with human male infertility. The sperm membrane is a key structure influencing sperm morphology and function in normal and pathological conditions. The fatty acid profile determines the performance not only of sperm motility but also of acrosomal reaction and sperm-oocyte fusion. This review presents available knowledge on the role of fatty acid composition in human sperm and spermatogenesis and discusses the influence of dietary fatty acids on the sperm fatty acid profile. Recent studies in biological sciences and clinical researches in this field are also reported. The topic object of this review has potential application in medicine by identifying potential causes of infertility.

## 1. Introduction

Fatty acids (FAs) esterified to phospholipids are part of the cell membrane and contribute to the structural components. In addition to being part of cell membrane phospholipids, FAs are an energy source and as precursors of bioactive lipid mediators have a strong influence on cellular responses and functions [1]. Balancing of lipid rafts and the release of secondary messengers [2] are involved in FA control of intracellular and extracellular signaling pathways in numerous types of cells and tissues [1]. Thus, FAs could contribute to and take part in disease incidence, severity, and outcome [1, 3, 4]. In particular, FAs have crucial roles in biophysical, biochemical, and signaling processes that act as sensing mechanisms and stimulus transduction, thus participating in epigenetic control pathways [5–7].

FAs are classified, according to the presence of double bonds in their chain structure, as saturated FAs (SFAs) in the absence of double bonds, as monounsaturated fatty acids (MUFAs) when a single double bond is present, and as polyunsaturated FAs (PUFAs) when having two or more double bonds. The position of double bonds in unsaturated FA is often written using the n-x notation where x indicates the position of the first carbon with a double bond counting from the methyl end of the chain (n-) [8, 9]. Membrane fluidity, flexibility, fusion, fission, and curvature largely depend on the FA composition of phospholipids [10]. Phospholipids with a high amount of PUFA will increase membrane flexibility due to the multiple double bonds, whereas saturated or *trans*-FAs would make it rigid [11].

It is well known that the Western diet is relatively poor in n-3 PUFAs and rich in n-6 PUFAs [12]. Because arachidonic acid (ARA, C20:4n-6) is a precursor to proinflammatory mediators, the role of an increased dietary intake of ARA, or of its metabolic precursor (linoleic acid (LA), C18:2n-6), in elevating the inflammatory process is debated [1, 13].

In this review, the role of FA composition in sperm quality and spermatogenesis efficiency, together with the effects of dietary FAs on the sperm FA profile, is reviewed. The relevance of FA composition to the quality of sperm parameters is discussed in light of the potential application in medicine by identifying potential causes of infertility.

## 2. Fatty Acids: Sources, Synthesis, and Function—Distribution in Reproductive Tissues

**Figure 1:**
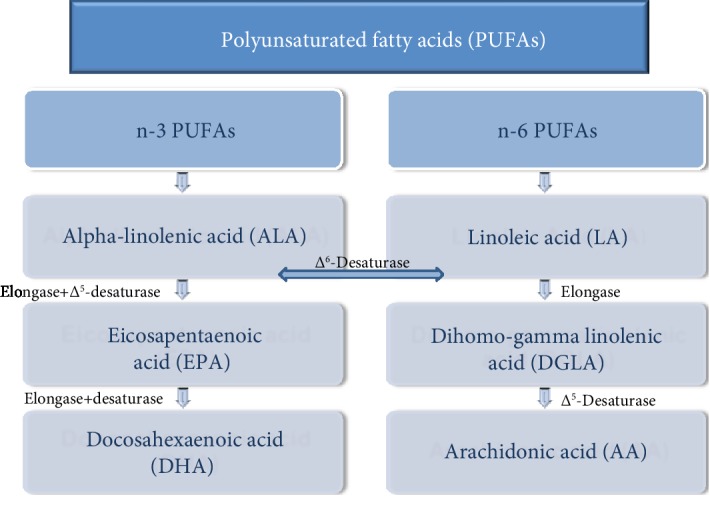
Summary of elongation and desaturation occurrence of polyunsaturated fatty acid (PUFA) metabolism.

Alpha-linolenic acid (ALA, C18:3n-3) and LA, respectively, as n-3 an n-6 PUFAs, are essential fatty acids (EFAs) that cannot be synthesized by animals and share the same elongation and desaturation process (Figure 1) in the metabolic pathway of PUFAs [14]. This process is catalyzed by elongating and desaturating enzymes, specifically elongases 2 (Elovl2) and 5 (Elovl5) and *Δ*^6^-desaturase (FADS1) and *Δ*^5^-desaturase (FADS2). FADS1 introduces a double bond to ALA and LA to extend into long-chain (LC) PUFA (LC-PUFA) [15, 16]. It is the rate-limiting enzyme in the synthesis of LA to ARA (Figure 1) and ALA to eicosapentaenoic acid (EPA, C20:5n-3) and docosahexaenoic acid (DHA, 22:6n-3) [17]. Furthermore, because n-6 and n-3 FAs compete for the same enzyme pathways, their metabolism is largely affected by the availability of the substrates and by the affinity of ALA and LA for the different enzymes. Moreover, the elongation/desaturation rate of EFAs (Figure 1) differs between species and within the same species, and it is affected by sex, hormonal status, intestinal biome, genetic strain, and feed [18].

Numerous studies have demonstrated that EPA and DHA are poorly synthesized in most animal species and in particular humans [19]. Notably, the LC-PUFA production in men is 15% lower than that in women [20]. The biosynthesis of LC-PUFAs also varies among human populations, and the levels of LC-PUFAs in African American men were found to be higher compared to European Americans [21]. These differences are linked to polymorphisms in the FADS gene leading to higher capacity to biosynthesis of LC-PUFAs from LA and ALA.

PUFAs and their metabolites act as secondary messengers in the cell membrane. In fact, after binding to growth factors and hormones and to membrane receptors thereafter, phospholipase A_2_ is activated and releases ARA, EPA, and DHA from the sn-2 position of the phospholipids. These molecules become substrates for eicosanoid biosynthesis: the ARA, via cyclooxygenases (COX), leads to the synthesis of 2-series prostanoids (prostaglandin E_2_, prostacyclin I_2_, and thromboxane A_2_), while the activity of lipoxygenases (LOX) on ARA generates 5-hydroperoxyeicosatetraenoic acid, which in turn produces the 4-series leukotrienes [12]. Moreover, EPA and DHA are converted by the same enzymes, e.g., COX and LOX, to 3-series prostanoids (prostaglandin E3, prostacyclin I3, and thromboxane A3) and 5-series leukotrienes, respectively [22]. These two classes of EFA metabolites are not interconvertible and are metabolically and functionally distinct, where they have opposite physiological functions; n-6 FA derivatives have prothrombotic, proaggregatory, and proinflammatory properties while the n-3 metabolites have anti-inflammatory, antiproliferative, and antiatherosclerotic activity [12]. As a consequence, the balance of EFAs is important for good equilibrium and function of several tissues and biological machinery.

Also, other types of metabolites originating from PUFAs were recently discovered, such as nonenzymatic free radical/ROS-mediated PUFA metabolites (i.e., isoprostanoids) which are known as the secondary products of lipid peroxidation. Lately, it has become accepted that these molecules not only serve as markers of oxidative damage but also exhibit a wide range of bioactivities [23].

Other mediators termed as specialized proresolving mediators (SPM) are metabolites derived from enzymatic oxidation of EFAs including DHA and EPA. In particular, resolvin D (RvD), protectins and maresins, and resolvin E are DHA and EPA derivatives, respectively [24]. Nevertheless, the interaction of n-3 and n-6 FAs and their lipid mediators in the context of inflammation is complex and still not properly understood [1].

The testes and sperm have a characteristic lipid composition that is highly enriched in PUFAs, predominantly docosapentaenoic acid (DPA, 22:5n-6) in rodents and DHA in humans [25] and other mammals [26–28]. LA and ALA together with their metabolites, EPA and DHA, are deposited in reproductive tissues and potentially influence the reproductive function and fertility. As reported above, in the sperm plasma membrane, lipid composition and the degree of PUFA unsaturation are relevant to the membrane fluidity, flexibility, and receptor function. Such features are largely involved in the membrane fusion events occurring in fertilization. Really, it would be taken into consideration that the lipid component of the spermatozoon membrane, as a part of the membrane microdomains (plasma membrane microdomains are involved in sperm motility, ability to penetrate the *zona pellucida*, and other capacitation-dependent changes), influences the membrane characteristics that are required for reaching and fusing with the oocyte. Additionally, it has been shown that the n-3 and n-6 PUFAs are essential for the reproductive activity, representing about 30% to 50% of the total FA amount in the membrane of mammal spermatozoa, and contribute to acrosome responsiveness [28].

In assessing the influence of PUFAs on male reproduction capability, the activity of metabolites generated from PUFAs (PUFA metabolism is reported above) should also be taken into consideration. On this point, prostaglandins and SPM are involved in the regulation of inflammation and infection, with these last ones being processes involved in affecting male fertility [25, 26]. Moreover, the skipped diene structure of the PUFA makes them susceptible to peroxidation and possibly alters the membrane characteristics. In this regard, reduction in human semen quality, as a consequence of smoking, infection, irradiation, varicocele, oligozoospermia, and drug exposure, has been linked to oxidative stress and lipoperoxidation [25].

## 3. Relevance of Fatty Acid Metabolism in Spermatogenesis

**Figure 2:**
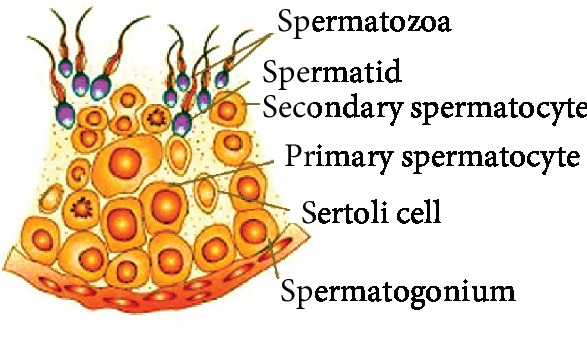
Spermatogenesis process: germ and Sertoli cells. The mitotic phase is represented by spermatogonia and the meiotic phase by primary and secondary spermatocytes and spermatids.

The process of spermatogenesis consists of a sequence of proliferative phases and differentiation and subsequent division to mitotic, meiotic, and spermatogenic stages (Figure 2). Each stage involves different cell types, including spermatogonia, spermatocytes, and spermatids, where lipid droplets increase throughout spermatogenesis [29]. Such phenomena demonstrate an intimate association between lipid metabolism alterations and fertility during spermatogenesis. FAs accumulate in testicular cells through two distinct processes: passive diffusion through the lipid bilayer and/or protein-facilitated transport mediated by CD36 glycoprotein, which is widely expressed in Sertoli cells [30].

The Sertoli cell, which is the supporting cell of spermatogenesis (Figure 2), has an important role in the endocrine and paracrine control of spermatogenesis. Functionally, it provides the cells of the seminiferous epithelium with nutrition, conveys mature spermatids to the lumen of seminiferous tubules, secretes androgen-binding protein, and interacts with endocrine Leydig cells. Throughout spermatogenesis, a dynamic and constant alteration in the membrane lipid composition of Sertoli cells occurs [31].

Liver and testicular cells convert dietary essential FAs (LA and ALA) to derivatives ARA, EPA, DPA, and DHA by alternating steps of elongation and desaturation [25]. As mentioned, these modifications include both *Δ*^5^- and *Δ*^6^-desaturases and elongases (Figure 1) specifically Elovl2 and Elovl5. Particularly, germ cells are known to be rich in PUFAs, more than the Sertoli cells, while the Sertoli cells are more active in converting the EFAs to DPA and DHA than germ cells [25]. This correlates well with the high expression of *Δ*^5^-desaturase and *Δ*^6^-desaturase in rat Sertoli cells and low expression in germ cells [32]. Human Sertoli cells can actively convert the 18-and 20-carbon PUFAs into their 22- and 24-carbon metabolites, and somehow, the conversion of n-3 FAs into 22- and 24-carbon metabolites is preferred by these cells over n-6 FAs in the metabolism, explaining to an extent the reason for high concentration of DHA in sperm [33].

In order to keep the energy of the seminiferous tubule at homeostasis, Sertoli cells react in response to several metabolic *stimuli*, through signaling cascades. For instance, the AMP-activated kinase is responsive to the global energetic status, the hypoxia-inducible factors are sensitive to oxygen concentration, and the peroxisome proliferator-activated receptors (PPARs) are influenced by FA availability in Sertoli cells. The development of metabolic diseases, including obesity and type II diabetes mellitus, induces these changes both directly and indirectly and as a consequence affects the Sertoli cell function and eventually male reproductive health [34].

During epididymal maturation, the lipid composition of the sperm membrane is remodeled, where the level of FA saturation is increased from the caput to the cauda epididymis, while the proportion of PUFAs remains similar along the epididymis [35]. The relative content of DHA is higher in epididymal versus testicular sperm in mice [36]. In addition, DHA is concentrated on the head or tail of the sperm and the levels vary among different species, where in human, the sperm head contains higher concentration of DHA [37]. If deficient, acrosome biogenesis is halted after the release of proacrosomal granules. It is further suggested that DHA is essential for the delivery of membrane protein misplaced syntaxin 2 for proper proacrosomal vesicle fusion [38].

The incorporation of PUFAs to semen extender is very crucial as it has different effects on semen quality for different animal species [28, 39]. Furthermore, seasonal differences in sperm FAs might in part explain the dismissal of equine spermatozoa for cryopreservation and cooled storage [40] in certain time of the year. Martínez-Soto et al. [41] suggested the spermatozoa and seminal FA profile as predictors of cryopreservation success in humans. n-3 PUFA, especially DHA, content in membrane FA was shown to have a direct association with sperm motility and viability after freezing/thawing, whereas MUFA abundance was inversely correlated with these sperm parameters. The subtemperature stress on the sperm demonstrates that the procedures may be designed to modify the lipid composition and/or antioxidant capacity of the ejaculate to make it more viable when cryopreserved.

The age of mammalians also modifies the sperm PUFA content. The proportion of PUFAs, namely, DHA, in the intact sperm, seminal fluid, and sperm head was lower in semen collected from mature bulls than that from young bulls. The finding indicates that age differentiates the rate of absorption and/or metabolism of PUFA that could influence spermatogenesis. Reduced proportions of major FAs in mature bulls might reduce membrane fluidity, which subsequently may affect the quality for cryopreservation and/or oocyte-sperm fusion through fertilization [42].

The role of FAs in spermatogenesis was confirmed also by studies on the enzymes involved in the FA metabolism. HELO 1 is an enzyme expressed in the testis and involved in the elongation of LC-PUFAs (ARA into adrenic acid (AdA) [43]).

Stearoyl-CoA desaturase 2 (SCD2) is the predominant *Δ*^9^-desaturase in the testis, and the Sertoli cells are the main site of its expression. Furthermore, both SCD1 and SCD2, as well as *Δ*^5^- and *Δ*^6^-desaturases, are highly expressed in the epididymis from sexually mature rats where the desaturase expression in Sertoli cells is hormonally regulated These desaturase enzymes can be induced by insulin, dexamethasone, and follicle-stimulating hormone [32].

In recent studies, lack of dietary n-3 PUFAs affected the spermatids. FADS2-KO mice fed with a PUFA-deficient diet except LA and ALA failed to produce mature spermatids and as a result created a defect on the acrosome formation [38]. Iizuka-Hishikawa et al. [11] reported that the loss of lysophosphatidic acid acyltransferase 3 caused a drastic reduction of DHA-containing phospholipids in mouse spermatids and led to excess cytoplasm around its head, which is normally removed by surrounding Sertoli cells via endocytosis in the final stage of spermatogenesis.

The sphingolipids of rodent spermatogenic cells (spermatocytes, spermatids) and spermatozoa consist of nonhydroxylated very long-chain and 2-hydroxylated very long-chain versions of VLC (C26 to C32) PUFAs that are not present in Sertoli cells. Recently, Santiago Valtierra et al. [44] investigated the role of elongase 4 (Elovl4) and fatty acid 2-hydroxylase (Fa2h), in rat testes with postnatal development and germ cell differentiation. Spermatocytes displayed the highest Elovl4 protein levels and activity. Fa2h mRNA was shown to be produced exclusively in germ cells, in particular round spermatids. Additionally, late spermatids, which result from elongation and head shape modifications, were shown to be enriched in Fa2h protein. For this reason, the distinctive expression of Elovl4 and Fa2h is associated with the abundance of n-V and h-V in the sphingolipid of rat spermatocytes and spermatids, respectively. Previously, Zadravec et al. [45] reported that the lack of Elovl2 was associated with a complete arrest of spermatogenesis, with seminiferous tubules displaying only spermatogonia and primary spermatocytes without further developing into germinal cells in mice.

Several hormones such as luteinizing hormone (LH) and adrenocorticotropin hormone (ACTH) may potentially change unsaturated FA composition in the testis by altering the activities of the enzymes [46]. In response to LH stimulation, together with increased testosterone secretion, the stored lipid is quickly depleted. Administering ACTH was prone to modification of *Δ*^5^-desaturase activity in testicular cells of normal mature rats. The total FA composition of the Sertoli cells isolated from ACTH-treated rats showed a significant increase in the relative percentage of LA and a decrease in 20- and 22-carbon PUFA biosynthesis suggesting an ACTH inhibitory effect on *Δ*^5^- and *Δ*^6^-desaturases [47].

Oxidative stress, as a serious damaging factor for male reproductive function, is particularly reputed to be a causative factor for male infertility due to its deleterious effects on the developing germ cells and sperm function [48]. In particular, free radicals and/or reactive oxygen species (ROS) are able to attack PUFAs in cell membranes altering their structure, function, and permeability. The injury induced by ROS in the germinal and testis cell membrane may lead to cell death, abnormality, and motility loss [49].

Certain oxidized PUFA metabolites, malondialdehyde [50, 51] and recently 4-hydroxynonenal [52], are suggested to be valuable biomarkers to monitor lipid peroxidation in sperm. Although useful, the application is problematic as it lacks specificity and sensitivity especially when utilized for *in vivo* measurements. Many of these limitations were resolved with the discovery of PUFA nonenzymatic oxygenated metabolites mainly isoprostanoids which are known as the intermediate products of lipid peroxidation.

Reports suggest that the type of diet potentially contributes to male fertility. Among the nutrients, supplemented carbohydrates and proteins do not have a remarkable effect on male fertility [53]. On the other hand, human and animal studies demonstrated that high intake of unsaturated, saturated, and *trans*-FAs inversely affected semen quality [54, 55].

Overall, during spermatogenesis, membrane remodeling takes place and cell membrane permeability and fluidity change. Lipids are important components of the germ cell membrane, where the volume and ratio fluctuate in different phases of spermatogenesis. Abnormal lipid metabolism can cause spermatogenic dysfunction and consequently male infertility. Furthermore, membrane lipids of germ cells are mainly composed of cholesterol, phospholipids, and glycolipids, which play critical roles in cell adhesion and signal transduction during spermatogenesis. In addition, retaining the membrane flexibility of the sperm tail is crucial for the sperm movement. High level of PUFAs in the sperm membrane assures higher fluidity of sperm cells, hence increasing the kinetic traits of the sperm [56–58].

An insight into the correlation of membrane lipid composition with spermatogenesis helps us better understand the mechanisms of spermatogenesis and provide new approaches to the diagnosis and treatment of male infertility. The sperm FA profile and the beneficial and detrimental effects of dietary FAs are the current focus of research in the field of nutrition and male reproduction. For all these purposes, the FA profile has been proposed as a marker of semen quality for patients with different semen parameters; this could be useful to obtain reference values that can be translated into the clinical practice [59].

## 4. Fatty Acids in Human Spermatozoa

Among the several studies on FA level in spermatozoa (Table 1), Zalata et al. [60] showed a comprehensive profiling of 26 different FAs, including SFAs, MUFAs, and n-6- and n-3-PUFAs. Most of the investigations included a limited number of patients and/or analyzed FAs in sperm [61–67], or FA content was compared between spermatozoa and seminal plasma [37, 63, 68]. In such comparisons, Zerbinati et al. [59] showed that DHA was 6.2 times higher in the corresponding isolated spermatozoa than in seminal plasma from normozoospermic samples, while myristic, palmitic, palmitoleic, vaccenic, linoleic, eicosadienoic, dihomo-*γ*-linolenic, and docosapentaenoic acids were about 2.0 times higher in spermatozoa compared to seminal plasma. In contrast, behenic, lignoceric, oleic, and mead acids were lower in spermatozoa compared to seminal plasma.

In human semen, about thirty FA molecular species were identified [59] ranging between SFAs, MUFAs, and PUFAs (n-6 and n-3 PUFAs), which have been shown to be specifically associated with sperm parameters. In particular, distinct FA compositions were related to specific seminal conditions (Table 2).

Several studies investigated sperm FA proportion comparing fertile and infertile subjects or normozoospermic and nonnormozoospermic individuals. Khosrowbeygi and Zarghami [69] measured elevated levels of palmitic, stearic, oleic, linoleic, arachidonic, and DHA in spermatozoa from patients with modified sperm parameters compared to normozoospermic subjects. Safarinejad et al. [70] found higher levels of n-6 PUFAs (LA and ARA) but lower levels of n-3 PUFA (ALA, EPA, and DHA) in spermatozoa and in blood plasma of infertile compared to fertile men. Other authors reported a lower seminal n-6/n-3 ratio in fertile men compared to the infertile ones, probably due to a significantly high amount of total n-3 PUFAs [41]. Recent investigation supports this observation where total n-3 PUFAs of normozoospermic individual's semen were significantly higher than those from men with oligozoospermia, asthenozoospermia, and oligoasthenozoospermia [71]. In addition, in normozoospermic subjects, it was shown that about 50%, 30%, and 20% of the total FAs were composed of SFAs, PUFAs, and MUFAs, respectively. Notably, four specific FAs (palmitic, stearic, and oleic acids and DHA) accounted for 74% of the total FA mass (palmitic acid, 24%; stearic acid, 22%; oleic acid, 16%; and DHA, 12%), and the single amount of DHA corresponded to 43% of the total PUFA content [59]. In this regard, in human sperm cells, DHA and palmitic acid were shown to be the predominant PUFA and SFA, respectively [37].

The amount of PUFAs, particularly DHA, in the sperm membrane augments as the sperm matures; it represents 20% of FA content in mature sperm compared with only 4% in immature germ cells [72]. Additionally, Haidl and Opper [73] reported a higher percentage of PUFAs in human sperm recovered from the cauda than in that recovered from the caput epididymis. Moreover, sperm DHA content has been positively correlated with sperm motility [63]. Many studies reported a high concentration of DHA in spermatozoa of normozoospermic subjects, but the concentration widely ranges from 4% to 30% [59, 65, 66, 74]. Calamera et al. [64] showed no differences in sperm DHA levels between normozoospermic and asthenozoospermic subjects.

Measurement of SFAs in seminal plasma showed that sperm concentration was positively correlated with palmitic acid but negatively correlated with stearic acid and elaidic acid [37, 68]. Seminal stearic acid was also negatively correlated with sperm motility [59]. Of the SFAs, palmitic acid was found to be the major type in human spermatozoa [60, 75]. Moreover, stearic acid but not palmitic acid was higher in oligozoospermic and asthenozoospermic subjects, compared to normozoospermics [59].

Discrepancies in the reported levels of FAs may be explained by differences in the methods of sperm preparation and/or method of measurement. Of note, it has been suggested that the dietary habits (both FAs and antioxidants) could deeply affect the FA profile of sperm [33].

Nonetheless, lifestyle and health status were suggested to affect FA sperm level; however, no association was found between the seminal FA profile and smoking habit [59], but negative correlations between the body mass index and levels of spermatozoon DHA and palmitic acid were reported [76].

To understand the role of the FA profile in male infertility, FA quality and quantity should be investigated in different pathological conditions such as anatomical or genetic abnormalities, systemic or neurological diseases, and infections. Varicocele remains the most common diagnosis seen in infertile men [77]. In this regard, a consistent reduction of DHA levels was found by Tang et al. [74] in infertile men with varicocele compared to fertile men, and Zerbinati et al. [59] observed that a group of patients with varicocele had a reduced number of sperm and motility with a modified seminal FA profile compared with the normozoospermic group. The varicocele group also showed significantly higher levels of elaidic acid, compared to normozoospermic individuals.

In summary, the data mentioned above make clear the relevant role of FAs in sperm function and suggest them as markers of sperm alterations. Our research group has pointed out the possible relation of FAs and sperm pathologies [78]. We investigated three groups of men: fertile, idiopathic infertility, and infertile with varicocele. Infertile men had higher levels of semen ROS than fertile men. High levels of semen ROS can cause sperm dysfunction, DNA damage, and reduced male reproductive potential [78]. Spermatozoa are susceptible to nonenzymatic oxidative damage because the plasma membranes are rich in PUFAs thus generating the prostaglandin-like end product known as isoprostanes (IsoPs). The infertile varicocele group, despite having a similar low sperm quality as idiopathic infertile patients compared to fertile men, had increased seminal levels of F_2_-isoprostanes (F_2_-IsoPs), a specific class of IsoPs, and high percentage of sperm immaturity; this suggests that an appropriate FA composition is needed for sperm maturation [79]. The association of sperm immaturity and high levels of seminal F_2_-IsoPs has been detected also in a patient carrier of round-headed sperm, a systematic sperm defect characterized by round nuclei with immature chromatin [80].

Compared to PUFAs, *trans*-FAs are associated with sperm quality in a different manner. Chavarro et al. [81] reported that semen levels of *trans*-FAs are inversely related to sperm concentration, and Zerbinati et al. [59] showed that oligoasthenozoospermic men had higher levels of seminal elaidic acid compared to normozoospermic subjects. As previously reported, they also found upregulated levels of elaidic acid in varicocele that could have deleterious consequences in these patients.

In conclusion, different FA contents in spermatozoa and seminal plasma have been described in individuals with pathological conditions compared to fertile men. It can be surmised that the FA profile could represent a good marker in male infertility and proper dietary integration of FAs may be a potential therapy for infertility.

## 5. Spermatozoa, Fatty Acids, and Diet

A recent metaregression analysis reported a significant decline in total sperm counts between 1973 and 2011 globally [82]. These data strongly suggest a notable decline in male reproductive health, with crucial implications for human reproduction and perpetuation of the species. Investigating modifiable lifestyle factors that influence human fertility is of major clinical and public health importance for understanding the problem [83]. Indeed, several observational studies that explored the associations between dietary patterns, food and nutrient consumption, and sperm quality suggest that adhering to a healthy diet (e.g., the Mediterranean diet) may improve male sperm quality parameters [84].

As noted, testis maturation, germ cell development, and function of sperm are related to lipid composition. PUFAs cannot be endogenously synthesized by humans and therefore must be obtained from food such as nuts, seeds, vegetable oils (source of LA and ALA), seafood (source of EPA and DHA), and meat and dairy (source of ARA). Dietary FAs influence the sperm FA profiles, and it appears to be the most sensitive to dietary n-3 PUFAs (ALA, EPA, and DHA) [33]. Consuming these foods modified the semen quality and FA sperm composition [33], whereas increased intake of SFAs or *trans*-FAs is reported to lower male reproductive ability in humans and animals [85]. Jensen et al. [86] observed that 701 young Danish men from the general population have a dose-response association between increased intake of saturated fat and decreased total sperm count and sperm concentration. In addition, a diet supplemented with fish oil increased DHA in the testis of rodents [87] and accumulated in the sperm membrane of humans [67]. In fertile individuals, administration for 4 weeks of high level of menhaden oil (50 ml) rich in DHA+EPA on a daily basis [88] had no effect on sperm motility, but semen phospholipid EPA increased.

Conquer et al. [89] studied serum and spermatozoon concentration of FAs and spermatozoon motility in asthenozoospermic men. In this double-blind, randomized, placebo-controlled study, both 400 and 800 mg/day DHA regimens increased serum DHA concentration but unaffected the spermatozoon DHA concentration and motility. In general, infertile men had lower concentrations of n-3 FAs in spermatozoa than fertile men [70] while it is suggested that oligoasthenoteratospermic men with low levels of EPA and DHA may benefit from n-3 FA supplementation [67]. After 32-week supplementation of 1.84 g of EPA (0.72 g) plus DHA (1.12 g) per day, oligoasthenoteratozoospermic men with low levels of EPA and DHA showed increased spermatozoon number, motility, and morphology, but the treatment had no effect on semen volume or serum sex hormone concentrations [67]. Supplementation with DHA+EPA (990 mg/d and 135 mg/d, respectively) for 10 weeks to healthy subjects demonstrated no effect on sperm parameters but prevented DNA fragmentation [90]. Esmaeili et al. [37] found improvement of male sperm parameters after 4 weeks of n-3 PUFA diet, and the response was time-dependent and dose-dependent. Recently, González-Ravina et al. [91] demonstrated the importance of DHA supplementation as a means of improving sperm quality in asthenozoospermic men.

Recent gene knockout strategies as well as analyses of human genetic disorders have unveiled several important molecules involved in the uptake and trafficking of DHA; however, the mechanism of how the lipid profile affects the male reproductive system is not well understood [11].

It should be underlined that the sperm membrane enriched of long-chain PUFAs is more prone to oxidation [92, 93] due to the numerous skipped diene formations in the structure. Accordingly, for a better response in the intervention studies, it is probably more effective to supplement the subjects with a strong antioxidant with high-dose PUFA to avoid unnecessary oxidation.

In general, an increased content of n-3 PUFAs is expected to influence the regulation of PPAR*γ*, apoptosis, eicosanoid formation, and hormone activity [37]. It has been reported that the inclusion of nuts in a Western diet significantly improved the total sperm count, vitality, motility, and morphology, and it was explained by the reduction in sperm DNA fragmentation [84]. *In vitro* and animal studies have shown that n-3 PUFAs are important substrates in early reproductive events, including improved fecundity, oocyte maturation, and embryo implantation [94, 95], and aid in restoring fertility and spermatogenesis in male rodents [96].

Several dietary studies related to PUFA supplementation have also demonstrated their capability to sustain sperm motility, viability, and fertility during chilling and freezing as well as improving testis development and spermatogenesis in a variety of livestock species [28]. In rats fed with a high-fat diet, olive oil (a source of MUFAs) and krill oil (a source of n-3 PUFAs) partially counteracted the negative effects of a high-fat diet and improved sperm quality, by increasing gamete motility, reducing oxidative stress, and slightly improving mitochondrial respiration efficiency [97]. Dietary supplementation with pomegranate seed, containing the PUFA punicic acid (18:3, n-5), in cloned goats improved sperm motility and viability following freezing-thawing and maintains developmental competency [98]. A diet enriched with vitamin E, zinc, selenium, folic acid, and n-3 PUFAs for at least two months improved sperm quantity and quality, especially sperm count and motility, and modified physical and functional properties of the sperm cell membrane in healthy dogs [57]. Studies on a rabbit model showed that the dietary supplementation of n-3 PUFAs and antioxidants [99, 100] largely altered the sperm membrane and improved the motility rate and the sperm speed. Moreover, rabbits treated with a diet supplemented with 10% of extruded flaxseed or 3.5% of fish oil showed a higher distribution of DHA and EPA in the testes and sperm membranes compared to controls [101].

In addition, in mice, either excessively high or insufficient n-3 PUFA consumption prior to conception until adulthood may cause adverse long-lasting effects on reproductive maturation and function of the progeny [102]. Finally, in the Seba's short tailed bat (*Carollia perspicillata*) concomitant to an increase in sperm velocity, the level of FA saturation increased from the caput to the cauda epididymis, while the proportion of PUFAs remained similar along the epididymis. Food treatments did not affect the sperm FA composition suggesting the presence of a specific endogenous mechanism [35].

Despite the relevance of n-3 PUFAs in male fertility, as reported above, it has been shown that PUFAs with 24–30 carbon atoms of the n-6 family in the testis are indispensable for normal sperm formation and fertility in male mice and that the investigated changes in n-6 fatty acid composition cannot be compensated by increased C22:6n-3 content [45].

Dietary fats may influence testicular function. However, most of the published literature on this field used semen quality parameters as the only proxy for testicular function. Minguez-Alarcón et al. [103] reported in healthy young Spanish men that MUFA intake was inversely associated with serum blood levels of testosterone and inhibin B whereas a positive association was observed between the intake of n-6 PUFAs and LH concentrations. In addition, the intake of *trans*-FAs was associated with lower testosterone. The intake of n-3 PUFAs was positively related to testicular volume while the intake of n-6 PUFAs and *trans*-FAs was inversely related to testicular volume.

Rats fed with an EFA-deficient diet developed testicular atrophy, and inclusion of LA did not prevent this incident and in fact they became infertile [104]. Separation of Sertoli cells and germ cells from rats fed with a fat-free diet for 9–14 days showed a shift in the lipid profile of both cell types towards a typical EFA deficiency pattern [105].

EFA deficiency has been associated with induced FA desaturase expression and activity in several tissues, but in the testis of sexually mature rats, none of the desaturases (SCD1, SCD2, *Δ*^5-^desaturase, or *Δ*^6^-desaturase) were induced in response to lowered contents of PUFAs [106]. This also applied to the caput epididymis, while EFA deficiency sensitivity was regained in the cauda epididymis; the desaturases were upregulated. A significant increase in the number of abnormal spermatozoa was observed in the cauda epididymis. It is suggested the alterations may be caused by the distortion of FA distribution in the spermatozoa, as well as in the epididymal tissue. On the other hand, a low-fat diet has been shown to decrease serum levels of androgens in human [107]. Thus, it cannot be excluded that the increased sperm abnormalities observed is an androgen-dependent effect induced by the diet [106].

Opposite to PUFAs, *trans*-FAs and SFAs appeared to have an effect on spermatogenesis. The association between *trans*-FAs, infertility, and fetal life has been reviewed by Çekici and Akdevelioğlu [108]. *trans*-FAs are found in commercially baked and fried foods, which accumulate in the testis, and high consumption is related to poor semen quality [33]. Previous studies reported [54, 55] that dietary *trans*-FA intake may be related to lower semen quality that eventually becomes linked to the ability of *trans*-FAs to inhibit the activity of desaturases and, as a consequence, limit the incorporation of LC-PUFAs into sperm membranes [109]. Eslamian et al. [53] conducted a case-control study to investigate the association of FA intakes and asthenozoospermia. They found that high intake of SFAs and *trans*-FAs was positively related to the odds of having asthenozoospermia. Dietary intake of n-3 PUFAs, but not of MUFAs and n-6 PUFAs, was inversely associated with asthenozoospermia.

Furthermore, dietary *trans*-FAs in the male are reported to decrease the chance of fertilization [110]. A study conducted on 141 couples undergoing assisted reproduction techniques reported that sperm from men with the highest *trans*-FA intake (1.20% of total energy intake) gave the lowest rate of fertilization. *trans*-FA intake correlated positively with low total testosterone and calculated free testosterone concentration but had a negative correlation with testicular volume [110] suggesting an effect on testicular function. Men in the top 25% of *trans*-FAs intake have been reported to have 37% lower total sperm count, 15% lower testosterone levels, and 4% less testicular volume than men with the lowest *trans*-FA consumption [103]. Similarly, *trans*-FA exposure in male mice caused *trans*-FA accumulation in the testes leading to lower serum testosterone concentrations and sperm count. Inhibition of spermatogenesis and testicular degeneration are severe reproductive disorders associated with *trans*-FAs in rodents [111, 112].

*trans*-FAs may affect semen quality that can also involve and influence PPARs. These have some similarity with steroid and thyroid hormone receptors, which are ligand-activated nuclear transcription factors. Both PPAR*α*- and PPAR*γ*-responsive genes are involved in lipid homeostasis, especially glucose and lipid homeostasis. *trans*-FAs inhibit the primary function of PPAR*γ* on sperm metabolism by downregulating PPAR*γ* mRNA expression. Such adverse effects of *trans*-FAs have been claimed to be responsible for infertility [37].

Given that lipids are composed of majority of the sperm plasma membrane, this information may open new possibilities for the development of male diagnostic tools [113].

## 6. Conclusions and Perspectives

As single molecules or as components of molecules, FAs play multiple biological roles ranging from participation in cell membrane composition to energy suppliers and signaling molecules [114].

FAs, available for cellular function and membrane composition, can derive from exogenous sources or *de novo* synthesis. In particular, dietary sources of ALA, DHA, and EPA are crucial to maintain an adequate supply in n-3 PUFA metabolism [114, 115].

**Figure 3:**
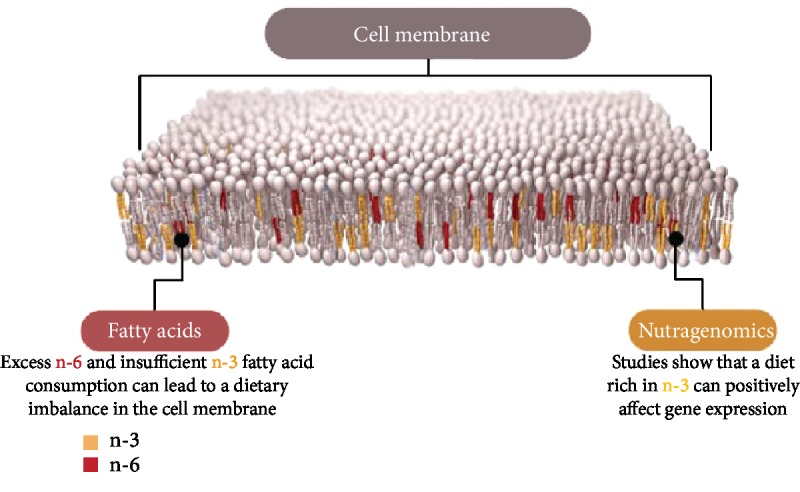
Incorporation of dietary n-3 and n-6 fatty acids in the cell membrane.

FAs, as a component of membrane lipid, are implicated in the modulation of biomembranes, and PUFAs heavily influence membrane permeability and elasticity (Figure 3). Thus, membrane PUFA composition plays a relevant role in different processes such as vesiculation, lipid flip-flops, and last but not least lipid-protein interactions. In particular, an increase in EPA and DHA is suggested to modify membrane stability and the composition in membrane-associated proteins by decreasing the MUFA/PUFA ratio. Such effects could be linked to the relevance of hydrocarbon chain length or *trans*-/*cis*-double bond and maintenance of the lipid array. Moreover, FAs work as energy suppliers and storage of lipophilic compounds. Both of these lipid features are connectable to cellular and spermatic biology [116].

Spermatogenesis is a complex process that involves the development of spermatozoa in the seminal tubules. The differentiation of spermatogonia into spermatozoa requires the participation of several cell types and the correct FA profile that contributes to a normal spermatogenetic process [117]. The importance of lipid composition, especially phospholipids, in the plasma membrane and semen plasma for spermatozoon function has since long been recognized [72]. PUFA level influences sperm maturation, motility, and acrosome reaction [118], and men with different seminal characteristics due to reproductive pathologies such as varicocele, infections, or others had shown different FA profiles [59]. Particularly, PUFAs may modulate oxidative stress, ROS production, and the inflammatory processes in spermatogenesis.

Sperm FA profiles and the beneficial and detrimental effects of dietary fatty acids are the current focus of research in the field of nutrition and male reproduction. In humans, diet is difficult to standardize, and research is mainly focused on the effect of dietary changes on male reproduction traits using an *in vitro* approach that does not take into account the dietary effect on spermatogenesis or on animal models.

Deep knowledge of how dietary lipid affects sperm lipid membrane composition, which in turn is relevant for sperm functionality, could improve the comprehension of sperm plasma membrane turnover and the susceptibility to oxidative damage. Such information will help to develop personalized nutraceutical treatments to improve male reproductive efficiency.

## Figures and Tables

**Table 1:** Sperm fatty acid levels in controls compared to abnormal seminal conditions.

Fatty acid category	Fatty acid	Controls	Infertile men	References
SFAs	Palmitic acid (C16:0)	19 (healthy)	26^∗^ OAT (mol% of total quantity)	Gulaya et al. [63]
		0.87 (controls)	1.92^∗^ AT, 4.44^∗^ OAT (nmol/10^6^ sperm)	Khosrowbeygi and Zarghami [69]
		26.4 (normozoospermic)	37^∗^ Asthen (nmol/10^8^ sperm)	Tavilani et al. [66]

SFAs	Stearic acid (C18:0)	17 (healthy)	8^∗^ Asthen (mol% of total quantity)	Gulaya et al. [63]
		13 (normal)	17^∗^ Asthen, 15.3^∗^Oligo (gr%)	Aksoy et al. [65]
		0.49 (controls)	1.05^∗^ AT, 2.71^∗^ OAT (nmol/10^6^ sperm)	Khosrowbeygi and Zarghami [69]
		4 (normal)	14^∗^ Asthen (nmol/10^8^ sperm)	Tavilani et al. [66]

MUFAs	Oleic acid (C18:1)	9 (normal)	11^∗^ Oligo (mol%)	Zalata et al. [60]
		11 (normozoospermic)	13^∗^ Asthen (wt% of total)	Conquer et al. [62]
		10 (normal)	11^∗^ Oligo, 11^∗^ OA (gr%)	Aksoy et al. [65]
		0.28 (controls)	0.41^∗^ Asthen, 0.65^∗^ AT, 1.67^∗^ OAT (nmol/10^6^ sperm)	Khosrowbeygi and Zarghami [69]

n-3 PUFAs	*α*-Linolenic acid (C18:3)	0.31 (fertile)	0.14^∗^ OAT (% of total fatty acids)	Safarinejad et al. [70]

n-3 PUFAs	Eicosapentaenoic acid (C20:5)	0.62 (fertile)	0.31^∗^ OAT (% of total fatty acids)	Safarinejad et al. [70]

n-3 PUFAs	Docosahexaenoic acid (C22:6)	9.5 (fertile)	6.55^∗^ OAT (% of total fatty acids)	Safarinejad et al. [70]
		21.5 (normal)	16^∗^ Oligo (mol%)	Zalata et al. [60]
		14 (normozoospermic)	8^∗^ Asthen (wt% of total)	Conquer et al. [62]
		16 (healthy)	5.3^∗^ Asthen (mol% of total quantity)	Gulaya et al. [63]
		25 (normal)	18^∗^ Asthen, 20^∗^ Oligo (gr%)	Aksoy et al. [65]
		0.84 (controls)	1.65^∗^ AT (nmol/10^6^ sperm)	Khosrowbeygi and Zarghami [69]
		32 (normal)	17^∗^ Asthen (nmol/10^8^ sperm)	Tavilani et al. [66]
		22.4 (normozoospermic)	17.5^∗^ Asthen, 15.3^∗^ Oligo, 13.6^∗^ OA	Martínez-Soto et al. [41]

n-6 PUFAs	Linoleic acid (C18:2)	3.1 (fertile)	5.22^∗^ OAT (% of total fatty acids)	Safarinejad et al. [70]
		0.2 (controls)	0.48^∗^ AT (nmol/10^6^ sperm)	Khosrowbeygi and Zarghami [69]
		8.4 (normal)	4.4^∗^ Asthen (nmol/10^8^ sperm)	Tavilani et al. [66]

n-6 PUFAs	Arachidonic acid (C20:4)	1.76 (fertile)	3.18^∗^ OAT (% of total fatty acids)	Safarinejad et al. [70]
		0.2 (controls)	0.40^∗^ AT, 0.65^∗^ OAT (nmol/10^6^ sperm)	Khosrowbeygi and Zarghami [69]

SFAs: saturated fatty acids; MUFAs: monounsaturated fatty acids; PUFAs: polyunsaturated fatty acids; Asthen: asthenozoospermia; AT: asthenoteratozoospermia; OAT: oligoasthenoteratozoospermia; OA: oligoasthenozoospermia. ^∗^Significant difference between the controls and infertile group. Control definition and units of measure are reported as indicated in the related reference sources.

**Table 2:** Seminal fatty acid levels in normozoospermia compared to abnormal seminal conditions.

Fatty acid category	Fatty acid (common name and number of carbons and double bonds)	Fatty acid contents^§^			
		Normozoospermia	OAT	AT	Varicocele
SFAs	Myristic acid (C14:0)	0.42	0.23^∗∗^	0.26^∗∗^	0.27^∗∗^
	Palmitic acid (C16:0)	23.97	18.98^∗∗^	19.58^∗∗^	20.67^∗∗^
	Stearic acid (C18:0)	22.05	25.53^∗∗^	24.30^∗∗^	23.92^∗∗^
	Arachidic acid (C20:0)	1.16	1.58^∗∗^	1.53^∗∗^	1.26
	Behenic acid (C22:0)	0.71	0.29	0.05	0.05^∗∗^
	Lignoceric acid (C24:0)	0.08	0.1	0.09	0.07
	Cerotic acids (C26:0)	0.09	0.10	0.09	0.12

MUFAs	Myristoleic acid (C14:1n-5)	0.12	0.23	0.11	0.18
	Palmitoleic acid (C16:1n-7)	0.34	0.24^∗∗^	0.25^∗∗^	0.28
	Vaccenic acid (C18:1n-7)	3.39	3.40	3.20	3.61
	Oleic acid (C18:1n-9)	16.61	22.44	19.21	17.53
	Gondoic acid (C20:1n-9)	1.40	2.39^∗∗^	2.04^∗∗^	1.84^∗^
	Erucic acid (C22:1n-9)	0.05	0.41^∗∗^	0.43^∗∗^	0.45^∗∗^
	Nervonic acid (C24:1n-9)	0.28	0.23	0.06	0.05

n-3 PUFAs	*α*-Linolenic acid (C18:3)	0.32	0.48	0.40	0.41
	Eicosatrienoic acid (C20:3)	0.11	0.15	0.09	0.07
	Eicosapentaenoic acid (C20:5)	0.40	0.21	0.11	0.11
	Docosapentaenoic acid (C22:5)	0.72	0.43	0.65	0.61
	Docosahexaenoic acid (C22:6)	12.82	3.42^∗∗^	7.67^∗∗^	8.33^∗∗^

n-6 PUFAs	Linoleic acid (C18:2)	3.59	3.22	3.67	3.67
	*γ*-Linolenic acid (C18:3)	0.05	0.13	0.11	0.12^∗∗^
	Eicosadienoic acid (C20:2)	0.65	0.63	0.81	0.79
	Dihomo-*γ*-linolenic acid (C20:3)	4.11	3.54	4.07	4.11
	Arachidonic acid (C20:4)	4.77	5.59	5.51	5.23
	Docosadienoic acid (C22:2)	0.10	0.20^∗^	0.16^∗^	0.18^∗^
	Adrenic acid (C22:4)	0.85	0.93	0.91	0.93
	Osbond acid (C22:5)	0.84	1	1.01	1.05

SFAs: saturated fatty acids; MUFAs: monounsaturated fatty acids; PUFAs: polyunsaturated fatty acids; AT: asthenozoospermia; OAT: oligoasthenoteratozoospermia; controls compared to AT, OAT, and varicocele (^∗^*P* < 0.01, ^∗∗^*P* < 0.001). ^**§**^Fatty acid contents are reported as percentage of total FAs by weight for samples of whole seminal fluid and are referred to Zerbinati et al. [59].

## Abbreviations

FAs: Fatty acids
SFAs: Saturated FAs
MUFAs: Monounsaturated FAs
PUFAs: Polyunsaturated FAs
DPA: Docosapentaenoic acid
DHA: Docosahexaenoic acid
LA: Linoleic acid
ALA: Alpha-linolenic acid
EFAs: Essential fatty acids
EPA: Eicosapentaenoic acid
ARA: Arachidonic acid
Elovl: Elongation of very long-chain fatty acid protein
*Δ*^5^-Desaturase: Delta-5 desaturase
*Δ*^6^-Desaturase: Delta-6 desaturase
*Δ*^9^-Desaturase: Delta-9 desaturase
FADS1: Fatty acid desaturase 1
FADS2: Fatty acid desaturase 2
COX: Cyclooxygenases
LOX: Lipoxygenases
SCD: Stearoyl-CoA desaturase
LH: Luteinizing hormone
ACTH: Adrenocorticotropin hormone
ROS: Reactive oxygen species
*trans-*FAs: *trans*-Fatty acids
PPAR: Peroxisome proliferator-activated receptors.
